# Foot Strike Pattern Detection Using a Loadsol^®^ Sensor Insole

**DOI:** 10.3390/s25144417

**Published:** 2025-07-15

**Authors:** Keiichiro Hata, Yohei Yamazaki, Misato Ishikawa, Toshio Yanagiya

**Affiliations:** 1 Faculty of Physical Education, International Pacific University, Okayama 709-0863, Japan; 2 Institute of Sports Sciences, International Pacific University, Okayama 709-0863, Japan; 3 Graduate School of Health and Sports Science, Juntendo University, Chiba 270-1695, Japan; 4 Collaborative Research Program of Sports Facility and Environment, Juntendo University, Chiba 270-1695, Japan; 5 Institute of Health and Sports Science & Medicine, Juntendo University, Chiba 270-1695, Japan; 6 Juntendo Administration for Sports, Health and Medical Sciences, Juntendo University, Chiba 270-1695, Japan

**Keywords:** running biomechanics, plantar force, sensor insole, impact-related running injury prevention

## Abstract

**Highlights:**

**What are the main findings?**

- Foot strike pattern detection using the loadsol^®^ sensor insole method achieved high detection accuracy for rearfoot (94.7%) and forefoot (81.8%) strikes compared to a traditional kinematic approach.
- Runners exhibited mixed foot strike patterns, even at constant treadmill speeds, highlighting individual variability in running biomechanics.

**What is the implication of the main finding?**

- The loadsol^®^ sensor insole shows potential for field-based, step-to-step monitoring of FSP and impact forces, aiding injury prevention and performance analysis.

**Abstract:**

Understanding the foot strike pattern (FSP) and impact force of running-related injuries is crucial for athletes and researchers. This study investigated a novel method for detecting FSP using the loadsol^®^ sensor insole during treadmill running. Twelve collegiate athletes ran at three different speeds (12, 15, and 20 km/h), with their FSP determined using both the kinematic method based on the foot strike angle and the loadsol^®^ method based on the plantar force applied to the rear-, mid-, and forefoot sensor areas. This study provides significant insights into FSP detection. Comparing the kinematic method to the loadsol^®^ method, the rearfoot, midfoot, and forefoot strike detection rates were 94.7%, 37.1%, and 81.8%, respectively. Moreover, the FSP was not uniform, even during treadmill running at a constant speed, with most participants exhibiting mixed patterns across different speeds. The loadsol^®^ sensor insole could offer a promising device for in-field measurement of FSP and impact forces, potentially helping researchers and athletes better understand and predict the potential running-related injury risks by monitoring step-to-step variations in running biomechanics.

## 1. Introduction

Foot strike pattern (FSP) during running and changes in ground reaction force (GRF) as a function of changes in FSP are important biomechanical variables for predicting the potential risk of impact-related running injuries. FSP has been categorized into three patterns based on the initial foot contact during running: rearfoot strike (RFS), in which the heel makes contact with the ground first; forefoot strike (FFS), in which the ball of the foot makes contact with the ground first before the heel comes down; and midfoot strike (MFS), in which both the heel and ball of the foot make contact with the ground simultaneously [1,2,3]. Previous studies have reported that the first impact of the vertical GRF, which is used as an index of the impact force, is the difference between the RFS and FFS/MFS [1,4]. In addition, the partial load on the lower limbs was different depending on the FSP. Kulmala et al. [5] have reported that the patellofemoral contact force and stress were significantly higher in RFS than in FFS, although the plantar flexion moment and Achilles tendon force were significantly higher in FFS than in RFS. Moreover, a previous study using the ultrasound method clarified that the positive work on the Achilles tendon was greater in FFS than in RFS, suggesting that the partial load on the Achilles tendon or the muscle–tendon junction with the plantar flexors was greater in FFS than in RFS [6]. Therefore, the potential risk of running-related injuries around the knee joint was higher during RFS running, whereas the potential risk of running-related injuries around the ankle joint and Achilles tendon was higher during FFS running. Not only the magnitude of the impact force but also the interaction between the FSP and impact must be considered to understand the potential risk factors for running-related injuries. In addition, external factors, such as the gradient in an in-field environment, affect the FSP and impact force [7,8]. Devices that can measure and evaluate object variables in field and laboratory environments are necessary to further understand the causes of running-related injuries.

Conventional methods have been used to detect FSP and measure the impact force, including force platforms, motion capture systems, and sensor insoles that measure the center of pressure [3,4]. Although force platforms and motion capture systems can measure kinetic and kinematic variables with high accuracy, the measurement of natural running under various conditions is unsuitable because of the limited measurement environment. In addition, these measurement systems are unsuitable for measuring consecutive steps during overground operations. Hence, the potential risk factors for running-related injuries associated with the interaction between FSP and impact force may be overlooked. In contrast, the sensor insole is a powerful device not only for detecting FSP [3] but also for measuring plantar force [9].

In recent years, both hardware and analysis algorithms of sensor insoles for gait analysis and GRF estimation [10,11,12] have undergone significant development and improvement. These technologies are expected to be applied in a wide range of fields, including medicine, rehabilitation, and sports and health sciences. In addition, commercially available sensor insoles have been reported to measure force with high accuracy [9]. In particular, the loadsol^®^ is an insole sensor that is wireless and light, weighing approximately 16 g. The pressure sensors in the sensor insole were divided into three regions: forefoot, midfoot, and rearfoot. These regions corresponded to 40%, 30%, and 30% of the total insole length, respectively. This configuration of sensors is nearly identical to that used in previous studies to detect FSP, where the foot length was divided into three equal regions of approximately 33% each [3,4]. Although FSP was determined based on the center of pressure in previous studies [3], it is considered that identifying the time-varying plantar force acting on each sensor region may allow for a simpler and more accessible method of determining FSP using commercially available sensor insoles. Moreover, the loadsol^®^ sensor insole would possibly minimize the effect on the natural running style of the runner and would be a suitable device for measuring objective variables (i.e., step-to-step changes in the FSP and impact force) in an in-field environment.

Therefore, this study aimed to clarify FSP detection in the absence of GRF and motion capture data using the plantar force applied to the forefoot, midfoot, and rearfoot sensor areas of the loadsol^®^ sensor insole. We hypothesized that the FSP detected by the foot strike index was determined from the position of the center of pressure at the initial foot contact timing [3,4]. Hence, the FSP can be detected with high accuracy by measuring the magnitude and time variation of the plantar force on the forefoot, midfoot, and rearfoot areas of the sensor insole (Hypothesis 1). In particular, a plantar force equivalent to the body weight applied to the heel sensor area of the sensor insole reflected the distinct impact force of the total plantar force. Therefore, the method proposed in this study may be able to determine a typical bimodal waveform of the vertical GRF in the RFS. Thus, RFS and FFS/MFS could be accurately classified.

## 2. Materials and Methods

### 2.1. Participants

Healthy males and females who exercised including running regularly were recruited for this study. In addition, habitual FSP was assessed based on the participants’ self-reports, and the population included a mixture of RFS and FFS/MFS runners. A total of 12 collegiate students with a running exercise habit, including 7 males and 5 females (age: 22.1 ± 1.6 years; height: 1.68 ± 0.09 m; body mass: 62.7 ± 8.7 kg) participated in this study. Of these, the numbers of participants who self-reported as RFS and FFS/MFS were 5 and 7, respectively. All participants provided written informed consent. This study was approved by the local ethics committee of the institution and was conducted in accordance with the Declaration of Helsinki.

### 2.2. Experimental Procedures

The most appropriate size of the sensor insoles (loadsol^®^ pro, 3-sensor HMF, NOVEL, Pittsburgh, PA, USA) was placed in each participant’s shoes. The loadsol^®^ sensor insole was prepared in three sizes (product types V, W, and X), which were compatible with US 6 to 7, 7 1/2 to 8 1/2, and 9 to 10, respectively. In addition, six retroreflective markers were placed on the ankle, heel, and metatarsophalangeal joints of both feet.

The participants ran on a treadmill at three speeds—12 km/h (slow), 15 km/h (medium), and 20 km/h (fast)—for 1 min with habitual FSP. Sufficient rest periods were allowed between the running tasks.

**Figure 1:**
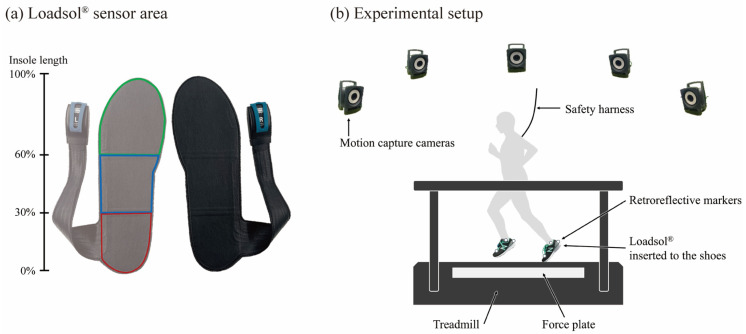
Loadsol^®^ sensor insole and experimental setup illustration. (**a**) Sensor area of loadsol^®^ defined based on insole length: rearfoot (green area, 30%), midfoot (blue area, 30%), and forefoot (green area, 40%). Transparency processing was applied only to the image of the left sensor insole. (**b**) Experimental setup showing the participant running on a treadmill with a loadsol^®^ sensor insole inserted into the shoes and a harness for safety. A force plate was embedded into the treadmill.

An overview of the loadsol^®^ sensor insole and the experimental setup is provided in Figure 1. The loadsol^®^ sensor insole used in this study can measure the plantar force at 200 Hz in the front, mid, and heel sensor areas, which were separated by 40%, 30%, and 30% of the insole length, respectively. The GRF was recorded at a sampling frequency of 1000 Hz from an instrumented treadmill incorporating two split-force plates (FTMH-1244, Tec Gihan Co, Ltd., Kyoto, Japan). The three-dimensional position of the retroreflective markers was obtained using a motion capture system constructed with eight cameras (Nexus 2.3, Vicon Motion Systems Ltd., Oxford, UK) at a sampling frequency of 250 Hz. The *X*-, *Y*-, and *Z*-axes of the global coordinate system were defined in the medial–lateral, anterior–posterior, and superior–inferior directions, respectively. To synchronize the time of each measurement system, the force platform system, motion capture system, and light-emitting diodes were connected to a synchronization system (wireless LED synchronizer transmitter PH-150 and receiver PH-155, Q’sfix, Tokyo, Japan) using a cable wire. The electrical signal with a square-wave output from the synchronization system was recorded using a force platform and a motion capture system. Moreover, light-emitting diodes that lit up in exact timing with the electrical signal output were recorded using the loadsol^®^ measurement application. The 30 consecutive steps were analyzed for each speed condition, and the total sample size obtained was 1080 steps (12 participants × 30 steps × three speed conditions).

### 2.3. Data Analysis

The GRF was smoothed using a fourth-order low-pass Butterworth filter at 30 Hz. The foot contact timing was defined from the obtained GRF or loadsol^®^ data, and the threshold value for initial foot contact and toe-off timing was set to 40 N. The coordinate data of the retroreflective marker positions were smoothed using a fourth-order low-pass Butterworth filter. The optimal cutoff frequency for each marker was identified using residual analysis [13] and ranged from 13.6 to 22.5 Hz.

FSP was determined using two different methods: (1) from the foot strike angle (FSP_FSA_), which is the gold-standard method [3], and (2) from the data of loadsol^®^ (FSP_loadsol_).

1. FSP_FSA_: The foot strike angle (FSA) at the initial foot contact during running was calculated as the angle between the vector from the heel to the metatarsophalangeal markers and *Y*-axis of the global coordinate system. FSA at initial foot contact was subtracted from the value in the static position [3]. According to a previous study [3], FSP_FSA_ was determined as FFS, MFS, and RFS when FSA < 1.68°, 1.68° < FSA < 8.08°, and 8.08° < FSA, respectively.
2. FSP_loadsol_: Loadsol^®^ could not measure the center of pressure owing to the limited number of pressure sensors. However, the FSP could be determined by the three pressure sensors in the rear-, mid-, and forefoot areas of the insole. FSP_loadsol_ was defined as RFS, MFS, and FFS when the plantar force exceeded the body weight first in the heel, midfoot, or forefoot area during the first half of the stance phase, respectively.

All the kinematic and kinetic data were analyzed using custom MATLAB scripts (MATLAB R2021b, MathWorks, Natick, MA, USA). To check the accuracy of FSP_loadsol_, the agreement rate and number of detected FSP_loadsol_ to FSP_FSA_ was compared at a certain running speed.

## 3. Results

**Figure 2:**
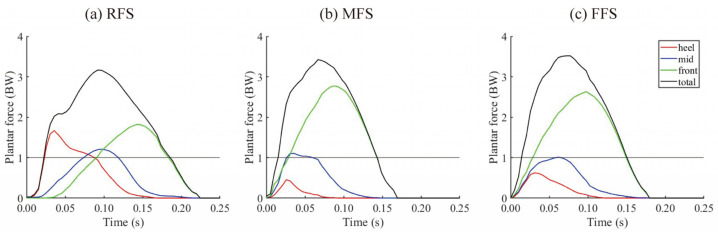
Plantar force during the stance phase in RFS (**a**), MFS (**b**), and FFS (**c**) running detected by loadsol^®^ data. Plantar force was measured in the heel (red), mid (blue), and front (green) sensor areas of loadsol^®^, and total plantar force (black) was calculated. The horizontal solid lines indicate the body weight equivalent to the plantar force. Plantar forces were normalized to body weight (BW). GRF, ground reaction force; RFS, rearfoot strike; MFS, midfoot strike; FFS, forefoot strike.

Typical results of the comparison of the plantar force obtained from the rear-, mid-, and forefoot sensor areas and total force from the loadsol^®^ during the stance phase in RFS, MFS, and FFS running are shown in Figure 2. The specific plantar force applied to the rear-, mid-, and forefoot sensor areas of the loadsol^®^ was observed at the given FSP.

**Figure 3:**
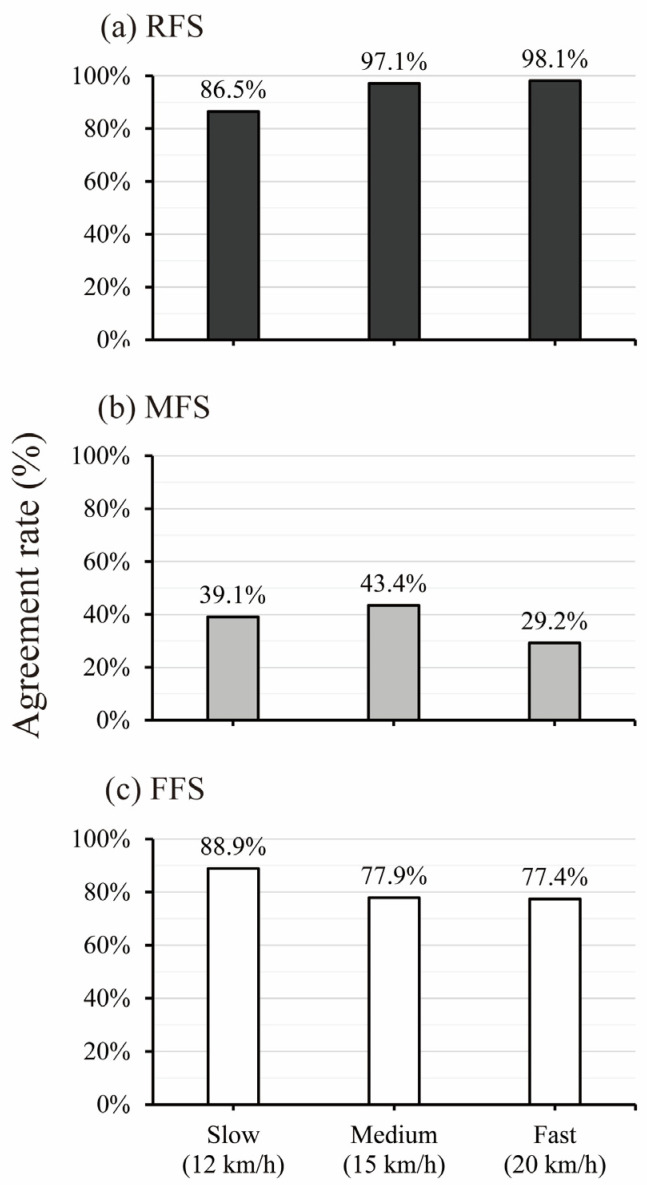
Agreement rate between the FSP_FSA_ and FSP_loadsol_ in each running speed condition. (**a**) RFS, rearfoot strike; (**b**) MFS, midfoot strike; (**c**) FFS, forefoot strike.

Figure 3 and Table 1 indicate the agreement rate between the FSP_loadsol_ from the plantar force applied to each sensor area and the FSP_FSA_ from the kinematic method across each running speed condition. At all speeds, the FSP_loadsol_ method was confirmed to have a detection accuracy of 94.7% for the RFS (slow speed, 86.5%; medium speed, 97.1%; and fast speed, 98.1%) and 81.8% for the FFS (slow speed, 88.9%; medium speed, 77.9%; and fast speed, 77.4%). The detection accuracy of the MFS was 37.1% (slow speed, 39.1%; medium speed, 43.4%; and fast speed, 29.2%).

FSP_FSA_ is the foot strike pattern calculated by foot strike angle, which is the gold-standard method; FSP_loadsol_ is the foot strike pattern calculated using the loadsol^®^ method; RFS stands for rearfoot strike; MFS stands for midfoot strike; and FFS stands for forefoot strike.

During the analysis steps of treadmill running at a constant speed, 10 of the 12 participants had a mixture of FSPs that were not uniform, including RFS, MFS, and FFS, at any speed. The only participants with a constant FSP were the two FFS runners (IDs A and K) (Table 2). Moreover, FFS and MFS were mixed in all five self-reported RFS runners, and RFS was mixed in five out of seven self-reported FFS/MFS runners (IDs C, E, H, I, and J).

## 4. Discussion

This study detected FSP based on the plantar force applied to the rear-, mid-, and forefoot sensor areas of the loadsol^®^ sensor insole to utilize it for in-field measurements. Assuming that the FSP_FSA_ is true, high accuracy of FSP_loadsol_ detection was shown in RFS and FFS. Although the accuracy rate was 94.7% for RFS and 81.8% for FFS under all speed conditions, the accuracy of FSP_loadsol_ detection in MFS was 37.1% (Table 1). Thus, detecting RFS and FFS with high accuracy was possible using only the three plantar force sensors on the rear- and forefoot regions based on the proposed new method using loadsol^®^ for determining FSP. The impact force depends on the FSP, and the partial load applied to the body varies [5]. The FSP was mixed even during treadmill running at a constant speed (Table 2), and the FSP during running would change in response to various external factors, even in field environments. Therefore, monitoring the interaction between FSP and impact force during running is essential in preventing running-related injuries and will help clarify the risk factors associated with running-related disorders in future research.

In the FFS and MFS detected by FSP_FSA_, it was a trend that the rate of RFS detected was high in the fast speed condition (Figure 3), with 11.7% of FFS and 51.5% of MFS detected as RFS based on the loadsol^®^ method (Table 1). Although a distinct impact force is not observed in FFS/MFS running at a common running speed [1,4], a distinct impact force appears in FFS/MFS sprinting, as reported in a simulation study [14]. The appearance of this distinct impact force may be attributable to an increase in the plantar force in the heel or midfoot sensor area, rather than in the forefoot sensor area. In FFS/MFS running under fast speed conditions, the running form would change to a sprinting-specific form as the running speed increased. Hence, plantar force applied to sensors other than the forefoot sensor area would be increased, reading to the increase in the ratio and number of RFS detections based on the loadsol^®^ method in this study.

**Figure A1:**
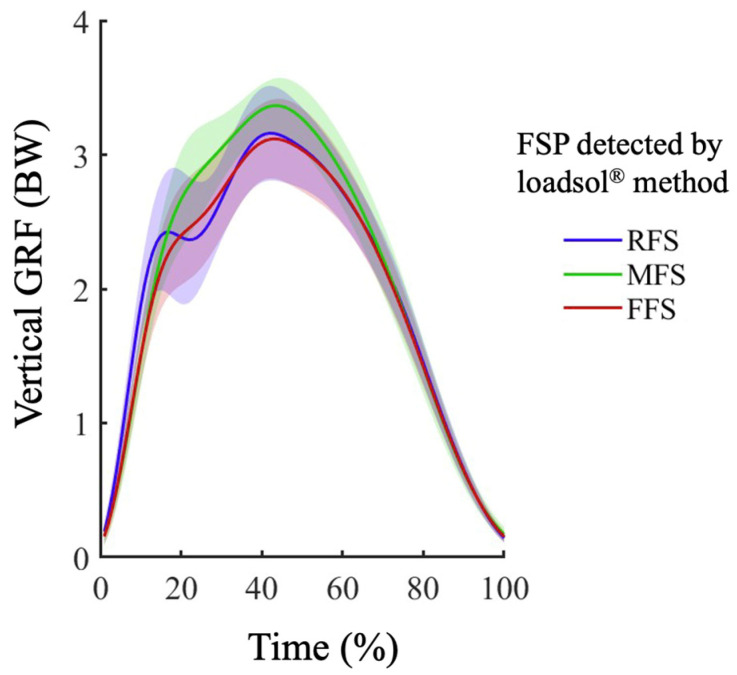
Unimodal and bimodal vertical GRF observed in MFS running. GRF, ground reaction force; FSA, foot strike angle; RFS, rearfoot strike; MFS, midfoot strike; FFS, forefoot strike; BW, body weight.

In the present study, although the accuracy of RFS and FFS detection was high, the accuracy of MFS detection was low at 29.2–43.4% under each speed condition, and MFS was detected as RFS or FFS with a similar detection rate (Table 1). The general definition of MFS is when the heel and ball of the foot make contact with the ground simultaneously [1,2,3]. Thus, even if the FSA was at a flat angle to the ground, the plantar force might have been applied to the rear or forefoot sensor area of the sensor insole rather than the midfoot sensor area, depending on the initial foot contact position and lower limb kinematics. In addition, MFS may cause a broad range of peak impact forces, from high to low [15], depending on ankle and knee joint compliance [1]. The FSP determined by the FSA method [3] and/or visual judgment of foot contact [2] may lead to misjudgment due to ankle adduction–abduction motion. Therefore, determining the FSP based on the loadsol^®^ method may be more appropriate. This would also be useful in examining impact-related running injuries. The vertical GRF waveform detected in the MFS by the FSP_FSA_ showed a mixture of the typical bimodal waveform in the RFS and the typical unimodal waveform in the FFS (Figure A1). In contrast, the FSP_loadsol_ determined by the loadsol^®^ method defined the step where a distinct bimodal vertical GRF waveform was observed as an RFS. The plantar force, which is equivalent to the weight applied to the heel sensor area, is reflected as a distinct impact force of the total plantar force (Figure 2a). Hence, the loadsol^®^ method could be used to determine the bimodal of the typical vertical GRF waveform in RFS, and it may be a suitable detection criterion for RFS. In many previous studies [16,17,18,19], FFS and MFS have been classified as a single group. Assuming that the detection of RFS by the loadsol^®^ is suitable, it would be recommended to categorize FSP into two types, RFS and FFS/MFS, when using loadsol^®^ for FSP measurement.

The proposed method for FSP detection in this study is considered applicable not only to treadmill running but also to in-field running. FSP would vary due to external factors, running speed, and fatigue. Observing FSP under these varying conditions and monitoring the interaction between FSP and impact forces on a step-to-step changing basis could be valuable for predicting running-related injuries. In future studies, the accumulation of data obtained using the present method, along with advances in big data analysis, may enable its application as a diagnostic support tool for running-related injuries in clinical settings.

This study has several limitations. First, the running test was performed on a treadmill and not in an in-field environment. Given that external factors, such as terrain slope, influence the FSP of running in in-field conditions, future research should investigate these effects more comprehensively. Second, the participants in this study included beginner runners, which may have contributed to the inconsistency observed in their FSP. However, the presence of runners who do not maintain a consistent FSP during running is a noteworthy finding, and this may provide valuable insights for the advancement of research on running-related injuries.

## 5. Conclusions

The proposed method could detect FSP_loadsol_ with high accuracy using the loadsol^®^ sensor insole. The results suggest that the proposed loadsol^®^ method was acceptable as a device that can be used to evaluate the FSP. Because the FSP was not uniform during treadmill running at a constant speed, step-to-step changes in the FSP and impact force need to be considered to clarify the potential risk factors for impact-related running injuries. Using the proposed loadsol^®^ method would lead to evaluating the partial loads on the lower limb depending on the FSP and elucidating potential risk factors for impact-related injuries.

## Figures and Tables

**Table 1:** Agreement and error rate and number (*n*) between the FSP_FSA_ and FSP_loadsol_.

	Speed			FSP_loadsol_	
			RFS	MFS	FFS
FSP_FSA_	Slow (12 km/h)	RFS (*n* = 74)	86.5% (*n* = 64)	12.2% (*n* = 9)	1.4% (*n* = 1)
		MFS (*n* = 133)	33.1% (*n* = 44)	39.1% (*n* = 52)	27.8% (*n* = 37)
		FFS (*n* = 153)	2.0% (*n* = 3)	9.2% (*n* = 14)	88.9% (*n* = 136)
	Medium (15 km/h)	RFS (*n* = 102)	97.1% (*n* = 99)	2.9% (*n* = 3)	0.0% (*n* = 0)
		MFS (*n* = 122)	21.3% (*n* = 26)	43.4% (*n* = 53)	35.2% (*n* = 43)
		FFS (*n* = 136)	0.0% (*n* = 0)	22.1% (*n* = 30)	77.9% (*n* = 106)
	Fast (20 km/h)	RFS (*n* = 106)	98.1% (*n* = 104)	0.0% (*n* = 0)	1.9% (*n* = 2)
		MFS (*n* = 130)	51.5% (*n* = 67)	29.2% (*n* = 38)	19.2% (*n* = 25)
		FFS (*n* = 124)	11.3% (*n* = 14)	11.3% (*n* = 14)	77.4% (*n* = 96)
	All speeds	RFS (*n* = 282)	94.7% (*n* = 267)	4.3% (*n* = 12)	1.1% (*n* = 3)
		MFS (*n* = 385)	35.6% (*n* = 137)	37.1% (*n* = 143)	27.3% (*n* = 105)
		FFS (*n* = 413)	4.1% (*n* = 17)	14.0% (*n* = 58)	81.8% (*n* = 338)

**Table 2:** Ratio and number (*n*) of FSPs during analyzed steps detected by FSP_loadsol_.

ID	Self- Reported FSP	Slow (12 km/h)				Medium (15 km/h)				Fast (20 km/h)		
		RFS	MFS	FFS		RFS	MFS	FFS		RFS	MFS	FFS
A	FFS/MFS	0% (*n* = 0)	0% (*n* = 0)	100% (*n* = 30)		0% (*n* = 0)	0% (*n* = 0)	100% (*n* = 30)		0% (*n* = 0)	0% (*n* = 0)	100% (*n* = 30)
B	FFS/MFS	33% (*n* = 10)	27% (*n* = 8)	40% (*n* = 12)		0% (*n* = 0)	60% (*n* = 18)	40% (*n* = 12)		47% (*n* = 14)	40% (*n* = 12)	13% (*n* = 4)
C	RFS	93% (*n* = 28)	0% (*n* = 0)	7% (*n* = 2)		100% (*n* = 30)	0% (*n* = 0)	0% (*n* = 0)		90% (*n* = 27)	0% (*n* = 0)	10% (*n* = 3)
D	FFS/MFS	0% (*n* = 0)	87% (*n* = 26)	13% (*n* = 4)		0% (*n* = 0)	73% (*n* = 22)	27% (*n* = 8)		0% (*n* = 0)	73% (*n* = 22)	27% (*n* = 8)
E	RFS	100% (*n* = 30)	0% (*n* = 0)	0% (*n* = 0)		100% (*n* = 30)	0% (*n* = 0)	0% (*n* = 0)		97% (*n* = 29)	3% (*n* = 1)	0% (*n* = 0)
F	FFS/MFS	3% (*n* = 1)	23% (*n* = 7)	73% (*n* = 22)		0% (*n* = 0)	33% (*n* = 10)	67% (*n* = 20)		0% (*n* = 0)	3% (*n* = 1)	97% (*n* = 29)
G	FFS/MFS	0% (*n* = 0)	57% (*n* = 17)	43% (*n* = 13)		0% (*n* = 0)	50% (*n* = 15)	50% (*n* = 15)		0% (*n* = 0)	50% (*n* = 15)	50% (*n* = 15)
H	RFS	87% (*n* = 26)	0% (*n* = 0)	13% (*n* = 4)		83% (*n* = 25)	10% (*n* = 3)	7% (*n* = 2)		97% (*n* = 29)	3% (*n* = 1)	0% (*n* = 0)
I	RFS	0% (*n* = 0)	0% (*n* = 0)	100% (*n* = 30)		50% (*n* = 15)	0% (*n* = 0)	50% (*n* = 15)		100% (*n* = 30)	0% (*n* = 0)	0% (*n* = 0)
J	RFS	53% (*n* = 16)	47% (*n* = 14)	0% (*n* = 0)		83% (*n* = 25)	17% (*n* = 5)	0% (*n* = 0)		100% (*n* = 30)	0% (*n* = 0)	0% (*n* = 0)
K	FFS/MFS	0% (*n* = 0)	0% (*n* = 0)	100% (*n* = 30)		0% (*n* = 0)	0% (*n* = 0)	100% (*n* = 30)		0% (*n* = 0)	0% (*n* = 0)	100% (*n* = 30)
L	FFS/MFS	0% (*n* = 0)	3% (*n* = 1)	97% (*n* = 29)		0% (*n* = 0)	40% (*n* = 12)	60% (*n* = 18)		83% (*n* = 25)	0% (*n* = 0)	17% (*n* = 5)

FSP, foot strike pattern; RFS, rearfoot strike; MFS, midfoot strike; FFS, forefoot strike.

## Data Availability

The data supporting the findings of this study are available from the corresponding author upon request.

## Abbreviations

The following abbreviations are used in this manuscript:

FSP	Foot strike pattern
RFS	Rearfoot strike
MFS	Midfoot strike
FFS	Forefoot strike
GRF	Ground reaction force
FSPFSA	Foot strike pattern determined by foot strike angle
FSPloadsol	Foot strike pattern determined by loadsol® data

## Appendix A

In the MFS running detected by FSA method, the typical unimodal and bimodal vertical GRF during the stance phase was observed. These waveforms represent the vertical GRF corresponding to RFS (blue), MFS (green), and FFS (red) as classified by the loadsol^®^ method. In other words, the FSA method classified bimodal vertical GRF patterns as MFS. However, the loadsol^®^ method provided a clear distinction of force waveform: bimodal patterns are identified as RFS (blue), while unimodal patterns are classified as MFS (green) or FFS (red).
